# Supplementary material for: Rolling Circle Amplification-Enabled Ultrasensitive Point-of-Care Test Method for Aflatoxin B1 in the Environment and Food
Source: Foods. 2024 Oct 7;13(19):3188. doi: 10.3390/foods13193188 (PMC11475565; doi:10.3390/foods13193188)
Supplement: Supplementary file 1 [file foods-13-03188-s001.zip › foods-3127708-supplementary.pdf]

Rolling Circle Amplification-Enabled Ultrasensitive Point-of-Care Test Method for Aflatoxin B1 in the Environment and Food

Hongyu Duan [1,†], Yuan Zhao [1,†], Xiaofeng Hu [1], Meijuan Liang [1], Xianglong Yang [1], Li Yu [1], Behrouz Tajdar Oranj [2], Valentin Romanovski [3], Peiwu Li [1] and Zhaowei Zhang [1,*]

[1] Oil Crops Research Institute of Chinese Academy of Agricultural Sciences, Wuhan 430062, China; dhy2634591222@163.com (H.D.); zhao_yuan2494@126.com (Y.Z.); xiaofenghu85@126.com (X.H.); liangmj@whu.edu.cn (M.L.); xianglongyang@foxmail.com (X.Y.); yuli01@caas.cn (L.Y.); peiwuli@oilcrops.cn (P.L.)

[2] Research Center for Environmental Determinants of Health (RCEDH), Kermanshah University of Medical Sciences, Kermanshah 67146, Iran; tajdar.tums@yahoo.com

[3] Center of Functional Nano-Ceramics, National University of Science and Technology MISIS, Moscow 101000, Russia; vramano@kth.se

* Correspondence: zwzhang@whu.edu.cn

† These authors contributed equally to this work.

S1. In vitro validation methods

Validation of RCA reaction system in tubes: the RCA reaction system contained 1 μM circular DNA, 1 mM dNTP, 0.5 units/μL phi29 DNA polymerase, 0.2 mg/mL BSA and 1 × phi29 buffer. First, 20 μL of the RCA reaction system was added to the final concentration of 1 μM biotinylated primer in a PCR tube at 37 °C for 0, 1, 2, and 3 h, respectively. After the RCA reaction, the tubes were incubated at 65 °C for 10 min. The RCA products were characterized by 12% native PAGE analysis. After electrophoresis analysis for 50 min at 120 V, the gel was submerged for 25 minutes in a solution containing GelRed dye to stain DNA lands and visualized under 302 nm UV light.

Verification of the RCA reaction in microwells: 10 μg/mL of streptavidin was preloaded on microwells. Meanwhile, after streptavidin was preloaded on microwells, we conducted the RCA reaction in microwells following the process described below. 50 μL of the RCA reaction system was added into a microwell at 37 °C for 3 h. After washing the microwells with PBST, 50 μL of the FAM-labelled signal probes (1 μM) were added to combine with RCA products and incubated at 37 °C for 1 h. Finally, after washing the microwells with PBST, the fluorescence intensity was recorded by using a SpectraMax i3x Microplate Reader at the excitation wavelength (498 nm) and emission wavelength (512 nm).

S2. HPLC parameters

The HPLC was K2025 (Wooking, China) equipped with photochemical derivation, and the chromatographic column was Agilent TC-C18 (4.6 mm × 150 mm, 5 μm). The mobile phase was 45% methanol, the running time was 12 min, the flow rate was 0.8 mL/min, the injection volume was 10 μL, and the column temperature was 35 °C. The excitation and emission wavelengths were 360 nm and 440 nm, respectively.

S3. Figures

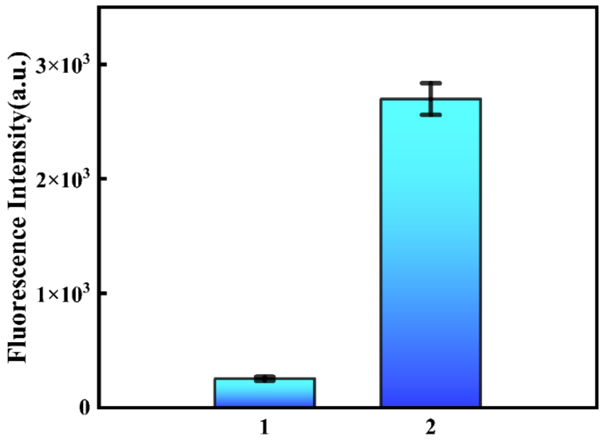

Figure S1. Feasibility of the RCA reaction with streptavidin preloaded on microwells. (1) Without biotinylated primer, (2) with biotinylated primer. (n=3)

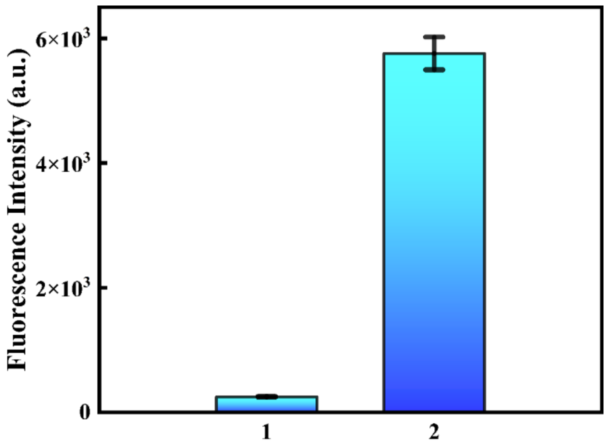

Figure S2. Feasibility of the RCA-SMIP on microwells. (1) Without biotinylated mAb, (2) with biotinylated mAb. (n=3)

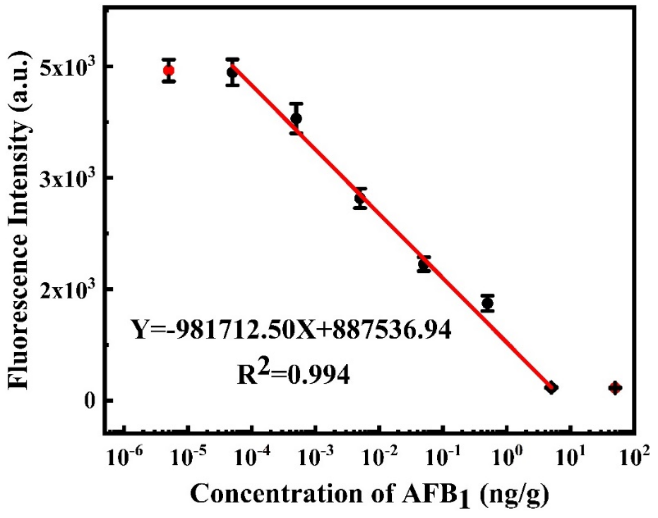

Figure S3. The corresponding linear calibration plots for AFB1 detection in peanut soil samples.

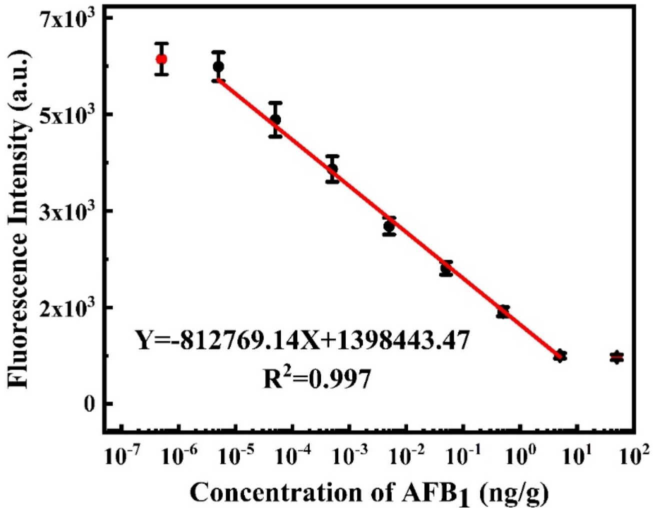

Figure S4. The corresponding linear calibration plots for AFB1 detection in irrigation water samples.
